# A Review on Bone Mineral Density Loss in Total Knee Replacements Leading to Increased Fracture Risk

**DOI:** 10.1007/s12018-017-9238-4

**Published:** 2017-10-27

**Authors:** M. Gundry, S. Hopkins, K. Knapp

**Affiliations:** University of Exeter Medical School, St Luke’s Campus, Heavitree Road, Exeter, EX1 2LU UK

**Keywords:** Total knee replacement, TKR, Fracture risk, Bone mineral density, BMD

## Abstract

The link between low bone mineral density (BMD) scores leading to greater fracture risk is well established in the literature; what is not fully understood is the impact of total knee replacements/revisions or arthroplasties on BMD levels. This literature review attempts to answer this question. Several different databases using specific key terms were searched, with additional papers retrieved via bibliographic review. Based on the available evidence, total knee replacements/revisions and arthroplasties lower BMD and thus increase fracture risk. This review also addresses the possible implications of this research and possible options to reduce this risk.

## Introduction

Two of the predominant pathological disorders that affect bone mineral density (BMD) are osteoarthritis (OA) and osteoporosis (OP). Due to the destructive nature of OA on the joints and the resulting loss of mobility and function, surgery is a primary option, and as such, OA accounts for between 80 and 90% of all total knee replacement (TKR/arthroplasty) procedures [1–5].

The relationship between OA and OP is complex and has been reported to be an inverse one [6], with OA being reported to increase BMD, and thus, it might be assumed to increase fracture protection. With such a high percentage of cases of TKR due to OA, it could be concluded that this protective effect would reduce fracture risk in TKR patients due to having higher BMD, but increased fracture rates have also been reported in OA. A narrative literature review was conducted to investigate the links between BMD, OA, and fracture risk in TKR; these specific key terms were searched in different combinations across the databases: PubMed, Google Scholar, Web of Science and Embase, with additional papers retrieved via bibliographic review.

## The Relationship Between BMD and OA

Research on the relationship between OA, BMD and the subsequent fracture risk has produced many controversial and conflicting results.

In 1972, Foss et al. were the first to observe the correlation between OA and fracture risk, concluding that patients with OA had a greater BMD for their age and thus had fewer hip fractures [6]. This suggested the possibility of a protective effect of OA due to higher BMD, with several studies supporting the link between OA and higher BMD (as shown in Table 1); Dequeker et al. in 2003 reviewed the relationship between increased severity of OA resulting in higher BMD scores, discovering 36 previous studies across 16 countries (Europe, the USA and Australia) covering a total of 37,774 subjects including 11,137 OA cases. Twenty-eight of these studies showed an increase in BMD with the remaining eight studies showing there was no increase in BMD [9].

**Table 1:** Studies showing BMD related to OA

Name of study	Participant numbers	Type of study	Type of OA if stated	Had OA	OA diagnosis	Comparator	BMD measurement site	Result
Arokoski et al. (2004) [7]	57	Not stated	–	27	Based on the American College of Rheumatology criteria regarding classification of OA in the hip	30 age matched controls	BMD and bone mineral content (BMC) measurements of the hip; femoral neck and head	Hip OA is not associated with an increase of BMD in the femoral neck or in the head of the femur
Chaganti et al. (2010) [8]	3923	Cohort study with cross-sectional analysis	Two distinct radiographic hip OA (RHOA) phenotypes: osteophytic and atrophic were examined	209 grade ≥ 2 185 grade 3–4	RHOA hip radiographs were assessed for five individual radiographic features of OA: joint space narrowing (JSN), osteophyte formation, cysts, subchondral sclerosis and femoral head deformity. Graded on severity 0–4	No RHOA (grade 0–1)	Lumbar spine, femoral neck, total hip and trochanteric sites	Both moderate and severe RHOA groups had significantly higher areal BMD at all BMD sites compared to the control group with no RHOA
Dequeker et al. (2003) [9]	37,774 across 36 papers	Meta-analysis	–	11,137	Not stated	26,637 controls	Not stated	28 papers showed an increase in BMD and 8 showed no increase
Hart et al. (2002) [10]	830	Cross-sectional	–	115 had baseline OA, of which 33 progressed to osteophytes after 4 years. Of the remaining 715, 95 had incident knee osteophytes within 4 years	Radiographic OA (ROA) on knee and hand x-rays, based on JSN and presence of osteophytes and thus graded on severity	No OA	Lumbar spine and hip	The 95 women with incident knee osteophytes had significantly higher baseline spine BMD and significantly higher hip than those without incident disease
Lethbridge-Cejku et al. (1996) [11]	649	Not stated	–	649 (240 grade 0, 186 grade 1, 187 grade 2, 35 grade 3, 1 grade 4)	Radiographs were read for features of OA using Kellgren Lawrence (KL) and reliable individual feature scales	No OA (grade 0–1)	Lumbar spine and hip	These results show that both men and women with radiographic changes of knee OA, specifically osteophytosis, have higher levels of adjusted spine but not hip BMD
Nevitt et al. (1995) [12]	4855	Cross-sectional	–	351 (grade 2), 228 (grade 3–4)	Radiographic OA on pelvis x-rays looking at hip features: osteophytes, JSN, subchondral sclerosis, cysts and femoral head deformity graded on severity	No OA	Femoral neck, trochanter, lumbar spine, calcaneus and distal radius	Elderly Caucasian women with moderate to severe radiographic hip OA had higher BMD in the hip, spine and appendicular skeleton than did women without hip OA

A study by Hart et al. [10] showed 95 women had a higher hip and spine BMD versus controls (0.79 versus 0.76 g/cm^2^, or 3.9%, and 1.01 versus 0.95 g/cm^2^, or 6.3%, for the hip and spine respectively); this itself is supported by other research [12] that concluded that OA resulted in higher BMD in the hip and spine, than women without hip OA. This trend was also seen in elderly men, who showed higher BMD in both the lumbar spine and hip compared to age-similar matched controls without OA [8]. This is further validated by research that shows an increase in BMD of the spine of patients with OA compared to controls [11, 13, 14].

There is research that states that although spine BMD might be high, hip BMD was not, as investigated by Lethbridge-Cejku et al. [11] who recruited 402 men and 247 women with OA. The results showed high levels of spine BMD but not hip BMD; this is supported by Arokoski et al. [7] whose findings suggest that hip OA is not associated with an increase of BMD in the femoral neck or in the head of the femur. It must be stated that there is also a small amount of research contrary to this that states OA lowers BMD compared to controls [15].

The majority of the research shows an increase in BMD in OA patients [6, 10–14, 16], but it must be acknowledged that there are other factors in OA; the phenotypes within OA such as osteophytic (which is osteophyte predominant [8]) and atrophic (which is joint space narrowing (JSN) predominant) both influence BMD scores. Out of the papers mentioned, only the Chaganti et al. study directly referred to the phenotypes’ impact; they showed significant differences in the hip and lumbar spine of areal BMD (aBMD) measurements for the two radiographic hip OA (RHOA) phenotypes compared to the control group. The osteophytic RHOA group had a higher aBMD at all sites compared to the control group: + 3.9% at the total hip (*p* = 0.002), + 8.5% at the femoral neck (*p* < 0.0001), + 4.6% at the trochanter site (*p* = 0.002) and + 7.2% at the lumbar spine (*p* = 0.0003). In contrast, the atrophic phenotype was not significantly associated with any difference in aBMD compared to controls [8]. The other studies mainly graded OA on a combination of factors resulting in a severity score; as such, these results might not be universal and may be more likely associated with the osteophytic phenotype. This is further supported by research that showed that obese patients have a more osteophyte dominant OA pattern compared to non-obese patients, 74.5% compared to 34.8% [17]; this coupled with increasing obesity in the population and the association of obesity with the onset and progression of OA in the knee [18] resulting in more TKRs might reflect the associated BMD changed and OA diagnosis.

Due to this recorded increase in BMD, it is generally thought that this would translate to having a protective effect on the bone by reducing fracture risk. This does not seem to be the case as several studies have argued against the protective nature of OA and actually demonstrate an increase in fracture risk (as shown in Table 2). Reasons for this contradiction in correlation could be due to limitations in the DXA scans only measuring two dimensions and not accounting for bone depth [25]. Furthermore, research has demonstrated that osteophytes contribute between 16.6 and 22% of the lumbar spine BMD variation in DXA scans in women and men respectively [26], possibly leading to an overestimation of higher BMD without the increase in bone strength; this might also explain the osteophyte phenotype predominance BMD results shown by Chaganti et al.

**Table 2:** Studies showing negative results between OA and fracture risk

Name of study	Participant numbers	Type of study	Had OA	OA diagnosis	Comparator	BMD measurement site	Result
Arden et al. (1996) [19]	937 (92 fractures)	Case-control study	319 with OA	OA was classified radiologically using standard x-rays of the pelvis, thoracolumbar spine, hands and weight-bearing knees. Radiographs were scored to the KL method	618 no OA	Lumbar spine and femoral neck	Despite having increased BMD of 5.3%, subjects with hip OA had a significantly increased risk of fracture compared to controls
Arden et al. (2006) [20]	6641	Randomised control trial	422 with OA and 277 with prevalent OA (clinically diagnosed)	The knee pain and OA questionnaire. They were also asked if they had ever received a clinician diagnosis of knee OA: “Has a doctor ever told you that you have OA of the knee?”	No knee OA 5774 (clinically diagnosed)	BMD not recorded	Patients with a clinical diagnosis of knee OA and with knee pain have an increased risk of non-vertebral and hip fracture
Bergink et al. (2003) [21]	4239	Cohort study	1466 fracture group contains 320 OA cases	ROA was assessed by means of the KL grading system in 5 grades (from 0 to 4)	2773 non-fracture group contained 675 OA cases	Lumbar spine and femoral neck	Although people with ROA had a higher BMD, their incident fracture risk was increased as compared with those without ROA
Chan et al. (2014) [22]	3864	Population-based prospective study	1077 with OA 325 fractures	The presence of OA was ascertained at baseline by self-reported diagnosis	1787 no OA 745 fractures	Lumbar spine and femoral neck	Overall, 29% of women and 26% of men had reported a diagnosis of OA. Fracture risk was significantly higher in women with OA than those without OA
Jones et al. (1995) [23]	1821	Longitudinal population-based study	462 with OA	Medication use and self-reported arthritis were assessed by a structured personal interview	1359 no OA	Lumbar spine and femoral neck	Individuals with self-reported OA, despite higher BMD, are not protected against non-vertebral osteoporotic fracture
Lee et al. (2014) [24]	1829	Cross-sectional study	34.20%	Radiographic knee OA was defined as KL grade ≥ 2	65.80%	Lumbar spine and femoral neck	In both sexes, the prevalence of vertebral fractures increased with age and was higher in the knee OA group than in the control group (in men, 13.2% in the OA group and 7.9% in the control group; in women, 27.7% in the OA group and 14.7% in the control group)

This bone strength argument is further supported by Lee et al. [24] whose cross-sectional study proposed that despite OA subjects having high systematic BMD, they were positively associated with vertebral fractures. Lee et al. suggested that bone quality, and consequently bone strength, may be decreased at the systemic level in knee OA, resulting in a higher risk of fracture [24]. A similar idea is shared by Ding et al. [27] whose research looked at OA in post-mortem participants, using microcomputed tomography scans of the microarchitecture of the proximal tibiae. This showed that medial OA trabecular bone was significantly denser but had poorer mechanical properties than normal bone. Ding et al. suggested that bone remodelling in OA leads to deterioration in architecture, resulting in poor quality bone, so although BMD could be retained, the bone quality was less, resulting in the possibility of greater fracture risk. This effect might be explained due to subjects with OA having a greater proportion of undermineralisation (immature matrix) in the bone [28]. This rationale is further supported by some research suggesting that bone trabecular microarchitecture was the key determinant of fractures in addition to the BMD data [29].

It must be stated that bone quality is affected by many other factors, not only the influence of OA. This includes factors such as obesity (which results in an increased risk of OA [22, 30]), diabetes and chronic kidney disease (CKD). Obesity is associated with higher BMD [31] but has been reported to lead to a lower rate of bone formation [32]. Type 2 diabetes is associated with higher BMD, but increased overall and hip fracture risk [33]. Research has shown that this might be due to changes in bone material properties rather than BMD, such as bone strength, structure, and quality, encompassing the microstructural and tissue material properties [34]. CKD can influence bone quality by altering bone turnover and mineralisation, resulting in microdamage and structural and material changes [35].

As shown in Table 2, several studies have argued against the protective nature of OA, reporting an increase in fracture risk despite subjects having increased BMD. One study reported a BMD increase of 5.3% compared to controls but no reduction in fracture risk [19]; this is further supported by the Rotterdam study [21] which utilised 2773 subjects and concluded that patients with knee OA had an increased risk of both vertebral (2.0-fold) and non-vertebral (1.5-fold) fractures. Furthermore, individuals with self-reported OA also had higher BMD but were not protected against non-vertebral osteoporotic fracture [23].

A study in 2014 [22] also demonstrated an increase in fracture rate among OA patients, in which 3864 subjects aged > 45 years were analysed. Results revealed that fracture risk was significantly higher in women with OA than those without OA. A prospective randomised control trial conducted by Arden et al. supports this argument where over 6500 men and women ≥ 75 years were recruited over 3 years, concluding that patients with knee pain and knee OA had an increased risk of non-vertebral and hip fracture [20].

It must be acknowledged that some studies have supported the idea of the OA protective effect; for example, Vestergaard et al. [36] conducted a case-control study using over 24,655 fractures matched for age and gender, with the main exposure being OA; their research showed that OA seemed to be associated with a decreased risk of fractures in multiple skeletal sites. This is agreed upon by Cumming and Klineberg [37] who had 189 participants (65–79 years old) with self-reported OA; the subjects with OA had fewer reported hip fractures than randomly assigned controls (4% compared to 13%). Additionally, Cumming et al. showed an inverse association between the number of joints reported to be affected by OA and the risk of hip fracture, with this protective effect being reported in both women and men [37].

Other research has shown a lack of any relationship between OA and fracture risk despite increased BMD [23, 38], with additional research using cohort studies showing no relationship between fracture risk and OA [39], contributing to the theory that OA does not have a protective effect on fracture risk.

Due to the contradiction and failure of the observed increase in BMD to translate into a protective effect and reduce fracture risk, several rationales were investigated. One rationale as to why an increased fracture risk may be explained, in part, was due to an increased fall tendency in patients with OA [38]. Research has shown that people with knee or hip OA have a greater number of falls and fracture risk compared to the general population [40, 41], even showing an increase in odds of falling correlated to the number of affected joints with OA [41].This theory is shared by other research [42], with some stating this is due to OA causing worsened postural stability and thus increasing the tendency to fall [23]. This is contradicted however by two cohort studies [20, 38] that reported that increased risk of fracture was independent of the number of falls. Although this in itself may be explained due to the severity of the falls and not the number of falls [20]. However, this rationale may be difficult to justify as fall data is often incomplete [43].

DXA bone density measurement provides an incomplete picture of bone strength as it is derived from the bone mineral content (BMC) divided by bone area, but does not account for the distribution of the trabeculae and the structural integrity of the microarchitecture. Bousson et al. created a tool called the trabecular bone score (TBS) [44] which is able to differentiate between microarchitectures that exhibit the same density [44]. This new method was investigated by Hopkins et al. [45] who recruited 19 post-menopausal women prior and post-TKR. The results exhibited that participants with TKR had higher mean lumbar BMD scores compared to controls but a lower TBS, suggesting that OA is potentially concealing poorer bone quality, even though it has a higher BMD. A further study by Hopkins et al. investigated differences in bone quantity and quality assessed by spine BMD and TBS [46]. These results demonstrated that the participants with TKR had higher BMD than the controls but poorer TBS scores [46].

TBS and BMD at the lumbar spine suggests that the generally higher BMD typically observed in OA patients may be disguising poor quality bone with less structural integrity [45]; this is supported by the rationale and results of the previous studies mentioned [24, 27] and might be the main reason that OA with high BMD does not a have protective effect in reducing fracture risk.

## BMD, Fracture and TKR Relationship

The relationship between low BMD and increased fracture risk is well recognised [47–52], but the association between BMD scores, TKR and the associated fracture risk is not well established.

The majority of the research shows a loss of BMD after a TKR (arthroplasty) (Table 3); Gazdzik et al. [54] reported a decrease in BMD 12 months after TKR surgery with the most significant BMD decrease during the period of 5–12 weeks after the surgery at the periprosthetic region. Other research concurs with this, stating the greatest loss of periprosthetic BMD has been observed within the first 3 months (12 weeks) after surgery [61–63], with some research reporting a temporary BMD loss of 13% at the proximal tibia [57].

**Table 3:** Studies showing BMD changes after knee arthroplasty

Name of study	Participant numbers	OA diagnosis if stated	Place BMD measured	Comparator	Intervals BMD measured	Result
Beaupre et al. (2015) [53]	97	–	Total hip and spine BMD	Pre-op BMD measurements	12 months post-operatively	Subjects undergoing primary cemented TKA had significant bone loss in the hip within 12 months, beyond that expected with age
Gazdzik et al. (2008) [54]	106	–	BMD measured in 4 regions around the knee	Baseline (first post op scan)	Before surgery, 2 weeks post-surgery (baseline), 5, 12, 24, 48 weeks	The most significant BMD decrease was observed in the period between 5 and 12 weeks after the TKA
Hopkins et al. (2016) [45]	62	28% of the controls had OA, and 86% of the testing group had OA	BMD measurements at the neck of the femur, total hip region and lumbar spine	46 age matched controls and non-operated hip	Pre-surgery baseline, and at 6 weeks, 6 months and 12 months post-surgery	Following surgery, an immediate loss of ipsilateral bone mass at the TH and NOF was demonstrated which was not restored at 12 months
Ishii et al. (2000) [55]	24 (31 knees, 47 hips)	All patients had the preoperative diagnosis of OA	BMD measurements of the hip	Non-operated hip	BMD measured preoperatively and a mean follow-up 48 months	Despite a predicted age-related loss of 4% during 2 years, 45% of the hips on the operative side and 59% of the hips on the non-operative side had BMD higher than preoperative levels. Of the hips, 81% on the operative side and 82% on the non-operative side had BMD that was within the expected 4% age-related loss
Kim et al. (2014) [56]	48	TKA was performed with patients who had a radiographic KL grade 3 or greater. Non-operative side had KL grade 2 for 40 patients and grade 3 for 8	BMD measured at femoral neck trochanter and total hip	Non-operated hip	Measurement of BMD was performed preoperatively and 1, 3 and 6 months and 1 year after unilateral TKA	Preoperatively, BMD of the femoral neck, trochanter and total hip on the operative side were lower than those on the non-operative side; however, there was no statistical difference
Li et al. (2000) [57]	28	–	BMD measured around proximal tibia measured at 9 regions	Baseline (first post op scan)	1 week after the operation (baseline); measurements were repeated at 3, 6, 12 and 24 months	The mean bone mineral density of all 9 regions of interest at the proximal tibia temporarily decreased by 13%
Liu et al. (1995) [58]	48	14 with knee OA	BMDs of both knees	20 age matched controls	Both knees were measured before operation and then at 3, 6 and 12 months after operation	The preliminary results demonstrate a significant progressive decrease of BMD in the distal femur of the operated knees after TKA, whereas the BMD of the non-operated knees remains stable
Petersen et al. (1995) [59]	25 (25 knees)	All had primary arthritis	BMD measured under the tibial component 6 regions	Baseline (first post op scan)	BMD measurements were performed within 2 weeks after the operation and at follow-up after 6 months and 1, 2 and 3 years	On average, the density for all regions of interest below the tibial component showed a significant and progressive decrease in BMD, reaching 22% at 3 years follow-up
Soininvaara et al. (2004) [60]	69	All patients had OA knees. The severity of OA was classified from preoperative standing x-rays using Ahlback’s classification	Hip and contralateral BMD measurements divided into regions	Non-operated side	At the time of operation (baseline) and at 1 year after operation	In all regions of interest, the mean baseline BMD of the affected side proximal femur was significantly lower than that of the contralateral side

This BMD loss is further supported by a study by Kim et al. [56] who investigated 48 Korean patients (11 males, 37 females, mean age 63 years) post-TKR; they reported a significant decrease in BMD at the trochanters and femoral neck in the first 3 months post-surgery, followed by a recovery of the BMD losses to − 2.14% at 12 months. A similar trend is seen across the research by Ishii et al. [55], Hopkins et al. [45] and Petersen et al. [59] who all reported a decrease in total hip BMD during the first 6 months post-operatively.

Other research investigated the effects of TKR 12 months post-operatively. Beaupre et al. [53] conducted a cohort study across 12 months and demonstrated that BMD decreased significantly by 1.80% at the total hip over that time. Soininvaara et al. [60] measured the BMD of bilateral hips in 69 patients undergoing total knee arthroplasty (TKA) (20 males, 49 females, mean age 67 years). They found a decrease in BMD at 12 months post-operatively of up to 2.7% per year in the ipsilateral hip and up to 1.18% per year in the contralateral hip; this bone loss affecting the operated side more than the non-operated has been seen in other studies [58]. Mintzer et al. [64] reported that within the first 12 months post-operatively, 68% of patients had radiographic evidence of bone loss at the distal anterior femur. There are many more studies that have shown a correlation between TKR (arthroplasty) and BMD loss [59, 65–70], although there are some studies that dispute this association and have shown no change in BMD post-TKR/A [57, 71, 72], with some research actually showing a small increase [73].

It is argued that BMD recovers to a baseline by 2 years [74], with research showing the greatest loss is within the first 2 years and eventually stabilising at that point [75], although this in itself has been contested [57, 76]. One explanation for this decline in BMD is a reduction in mobility of the patient post-surgery leading to reduced weight bearing and thus disuse related bone loss [64, 77]; this potentially explains the trend of such significant BMD reductions in the first 6 months and levelling out at 2 years post-operatively [55].

Due to the majority of papers reporting a significant loss of BMD post-TKR/A, the possible associated fracture risk must be investigated (this is shown in Table 4). A study by Meek et al. reported that women aged ≥ 70 years who had a TKR were 1.6 times more likely to have a fracture than younger patients and 2.3 times more likely to suffer a fracture than men [78]. This is further supported by Toogood et al. who stated that the greater majority of annual periprosthetic fractures were more often elderly and female [79]. Preliminary results from the Sahlgrenska Academy in Mölndal [80] analysed medical records from 1987 to 2002, concluding that individuals who had a TKR had an increased risk for hip fracture by 4%, with the risk for vertebral fracture increasing by 19% compared to the population without TKR.

**Table 4:** Studies showing fracture risk with TKR

Name of study	Participant numbers	Result
Lalmohamed et al. (2012) [42]	6763 cases (89 had knee arthroplasties (KA)), 26,341 controls (208 had a KA)	A 54% increased hip fracture risk was found in patients who underwent KA
Meek et al. (2011) [78]	44,511 primary TKRs and 3222 revision TKRs	Comparison of survival analysis for all primary and revision arthroplasties showed periprosthetic fractures were more likely in females, patients aged > 70 and after revision arthroplasty
Prieto-Alhambra et al. (2011) [43]	20,033 knee OA (cases) 100,065 (controls)	Hip fracture rates were non-significantly reduced compared with controls before the operation. In the year after TKR, risk increased significantly
Toogood et al. (2015) [79]	30,624 (total joint arthroplasty, TKA and total hip arthroplasty)	Individuals admitted with periprosthetic fracture were older, were more often female, were more often admitted emergently
Vala et al. (2016) [80]	The research followed the total Swedish population born 1902–1952 (*n* = 4,546,820) during the period 1987–2002 resulting in a total of 3719 having TKR and a fracture	After total knee replacement, the risk for hip fracture increased by 4% and the risk for vertebral fracture increased by 19% compared to the population without TKR

The Prieto-Alhambra et al. [43] research supports this increase in hip fracture after TKR reporting that hip fracture rates were insignificantly reduced compared to controls before the operation, but within 12 months post-operatively, TKR patients had a higher rate of hip fracture than controls, with relative risk increasing significantly up to 1.58 and then declining to equal the controls by 3 years. Additional research [42] has also shown a relationship between TKA and fracture risk, reporting a 54% increased risk of hip fracture, in particular among adult patients aged 71 years old, with the increase risk of hip fracture greatest after the first few years. Other research has shown an even higher figure, reporting a 58% increase in hip fracture in patients who had undergone TKR [43].

This increase in fracture risk during the first few years is time-dependent and as such could be associated with the evidence that supports early BMD decline as an important predisposing factor contributing to fracture risk [81–85].

There are other reasons put forward for the increased fracture risk in TKR patients, with some reports stating there is a higher incidence of falls thus a higher chance of fracture. Research by Matsumoto et al. [86] reported that of 81 patients who underwent TKA, the incidence of falls was 38% in the first year post-operatively, compared to 24% in the non-TKA cohort. Additional research also shows a higher rate of falls indicating scores of between 23 and 43% [87–90]. Although some research contradicts this and shows fall incidences were not significantly higher in the TKR group [45].

One possible intervention is antiresorptive treatment. Hahn and Won [72] investigated this, concluding that bisphosphate treatment just after TKR surgery prevented early BMD reduction in the hip and would be beneficial in the prevention of later hip fracture. This is supported by research by Carulli et al. [91], who proposed the use of bisphosphonate treatment in patients to not only prevent bone loss but increase implant survival. Other studies [92] reviewed the effectiveness of bisphosphonate use on post-TKR fracture risk recruiting patients who had received a TKR between 1986 and 2006 for knee OA. They concluded that bisphosphonate treatment after a TKR reduced the risk of fracture by 50–55%. Additionally, a meta-analysis [93] in 2015 reviewed the long-term effects in using bisphosphonates, reporting a significant decrease in implant revision after TKA or total hip arthroplasty (THA). Although caution should be utilised when administering bisphosphonates for long-term use, as research from 2017 has recently reported an increase in the size and number of microcracks leading to higher fracture risk in those patients on long-term bisphosphate use [94]. Furthermore, there have been reports of long-term use leading to increased atypical femoral fractures [95].

Some studies have looked at other possible antiresorptive treatments, such as hormone replacement therapy (HRT) to reduce BMD loss; several studies have shown an increase in BMD at all skeletal sites in early and late post-menopausal women [96, 97] with studies reporting a BMD increase of 5.3% at the lumbar spine and 7.6% at the femoral neck compared to 0.2 and 2.1%, respectively, in the control placebo group [98], although it must be noted that long-term use of HRT is strongly associated with breast cancer [99], so long-term administration is no longer advisable [100].

Other possible antiresorptive treatments are selective oestrogen receptor modulators (SERM); these have been reported to show an increase in BMD in the femoral neck by 2.1–2.4% and in the spine by 2.6–2.7% compared to placebo controls [101]. Unfortunately, there are obstacles to SERM such as causing menopausal symptoms (breast pain, hot flushes) and resulting in an increase in thromboembolic events [102, 103].

In contrast to antiresorptive treatments, there are also anabolic agents; these stimulate bone growth, with currently the only approved anabolic for systemic use being parathyroid hormone (PTH) [104]; this treatment stimulates osteoblasts, thus promoting increased BMD, although PTH has been associated with side effects such as headaches, dizziness, joint pain, depression [105] and hypercalcemia [106]. Another anabolic treatment investigated was strontium ranelate [104]; this treatment is capable of encouraging bone growth via stimulating osteoblast activity [107]. This drug was withdrawn in 2017 [108] due to safety concerns such as cardiovascular risks and increase risk of death [109].

Other research has investigated the selection of materials, design and alignment of joint components for replacements and how these might impact BMD [110, 111]. These mainly come in the form of metal implants and can be used in both tibial and femoral defects regardless of cementation.

These implants are primarily chosen due to being bioinert, able to support mechanical loads and being highly porous, promoting osteointegration [112]. Research has shown that tantalum cone implants were fully integrated after 2 years [113] and have been reported to maintain tibial bone density [114]; together with the material tantalum, porous titanium cones that mimic normal trabecular bone have been investigated; this type of implant has been shown to increase BMD at particular regions by 8.1% [115], with further research showing a similar favourable effect on BMD [116]. Some research however demonstrates that there is no significant difference in changes in BMD between the groups [117]. The titanium cone implants have also shown to have better stability than their tantalum counterparts [118]. In addition to the cones, some studies have investigated the effect of hydroxyapatite (a bioceramic that resembles the mineral constitutes of human bones and teeth [119]) coated onto the titanium implants to promote ingrowth; this combination has shown to increase shear strength [120] but has been reported to lead to decreased bone formation on porous coated titanium [121].

Metaphyseal sleeves have also been investigated; these come in a variety of different shapes and are press fitted into bone allowing bony ingrowth; research has reported excellent osteointegration and lasting fixation [122], showing ingrowth stability 3 months post-operation [123]. Unfortunately, there appears a lack of data about metaphyseal sleeves in TKR/A affecting BMD, with the main priority being stabilisation and survivorship.

## Conclusion

Low BMD has long been associated with an increased fracture risk, and the majority of the research shows that TKR results in reduced BMD, with potential increased fracture risk for the first 2 to 3 years. As such, it is suggested that patients with low BMD should be treated in order to combat this either through antiresorptive therapies, anabolic treatments or particular specialised implants prior to surgery in order to reduce this risk. The influence of low BMD does not only affect the patient; 60% of orthopaedic surgeons have stated that low BMD would influence their surgical plan and the implant design, yet only 4% performed BMD measurements preoperatively [124]. This is suggested by other research and states that surgeons must consider BMD loss if they are relying on bone for long-term stable fixation of the prosthesis [70].

In cases of patients with reported OA, their BMD measurements should utilise an additional type of measurement in addition to the DXA scan, either TBS or other means of assessment in order to assess bone architecture. TBS itself has started to be recognised and has been used in osteoporosis diagnosis with further suggestion for it to be used as a tool to monitor treatment effects [125]. This assessment would all be in addition to clinical risk factors that contribute to fracture risk independently of BMD including age, previous fragility fracture, premature menopause, a family history of hip fracture and the use of oral corticosteroids [126].

Finally, it must be noted, for the associated risks of fracture with TKR/A, the impact on quality of life for the patient is paramount. Research has shown that those undergoing a TKR due to high risk of knee OA have an improved quality of life compared to those who do not get a TKR [127, 128]. Furthermore, TKA has shown to have high patient satisfaction and long-term survival [129].
